# 

**Published:** 1902-06

**Authors:** 


CONTENTS.

| | PAGE |
|---|---|
| **Edward Long Fox, M.D. (Oxon.), F.R.C.P.** | 97 |
| **Bonville Bradley Fox, M.A., M.D. (Oxon.)** | 105 |
| **The Diagnosis of Oriental or Bubonic Plague.** By E. Klein, M.D., F.R.S. .. | 108 |
| **Two Cases of Localised Necrosis of the Lung, with Recovery.** By J. Michell Clarke, M.A., M.D., F.R.C.P. | 122 |
| **Some Cases of Ptosis.** By W. M. Beaumont, M.R.C.S., L.S.A. ... ... | 131 |
| **Diplococcal Bronchitis.** By P. Watson Williams, M.D. Lond. ... ... ... ... | 135 |
| **The Reputation of the Hotwells (Bristol) as a Health-Resort.** By L. M. Griffiths, M.R.C.S. Eng. ... ... ... | 142 |
| **Progress of the Medical Sciences:** | |
| **Medicine.** G. Parker, M.D., M.A. Cantab. | 153 |
| **Surgery.** C. A. Morton, F.R.C.S. ... | 157 |
| **Obstetrics.** W. C. Swayne, M.D. Lond. | 160 |
| **Reviews of Books** ... ... ... ... ... ... | 164 |
| **Editorial Notes** ... ... ... ... ... ... | 176 |
| **Notes on New Drugs and Preparations for the Sick** ... ... ... ... ... ... | 179 |
| **The Library of the Bristol Medico-Chirurgical Society** ... .. ... ... | 182 |
| **Meetings of Societies** ... ... ... ... ... | 186 |
| **Obituary** ... ... ... ... ... ... ... ... | 191 |
| **Local Medical Notes** ... ... ... ... ... | 191 |

